# Neuroprotective Function of DJ-1 in Parkinson's Disease

**DOI:** 10.1155/2013/683920

**Published:** 2013-05-16

**Authors:** Hiroyoshi Ariga, Kazuko Takahashi-Niki, Izumi Kato, Hiroshi Maita, Takeshi Niki, Sanae M. M. Iguchi-Ariga

**Affiliations:** ^1^Graduate School of Pharmaceutical Sciences, Hokkaido University, Kita-ku, Sapporo 060-0812, Japan; ^2^Graduate School of Agriculture, Hokkaido University, Kita-ku, Sapporo 060-8589, Japan

## Abstract

Parkinson's disease (PD) is caused by dopaminergic neuronal death in the substantia nigra, resulting in a reduced level of dopamine in the striatum. Oxidative stress and mitochondrial dysfunction are thought to be major causes of neurodegeneration in PD. Although genetic and environmental factors are thought to affect the onset of PD, precise mechanisms at the molecular level have not been elucidated. The *DJ-1 gene* is a causative gene for familial PD (*park7*) and also an oncogene. DJ-1 has various functions, including transcriptional regulation, antioxidative stress reaction, and chaperone, protease, and mitochondrial regulation, and its activity is regulated by its oxidative status, especially that of cysteine 106 (C106) of DJ-1. Excess oxidation of DJ-1, which renders DJ-1 inactive, has been observed in patients with sporadic PD and Alzheimer's disease, suggesting that DJ-1 also participates in the onset and pathogenesis of sporadic PD as well as familial PD. DJ-1 is also a stress sensor and its expression is increased upon various stresses, including oxidative stress. In this review, we describe functions of DJ-1 against oxidative stress and possible roles of DJ-1 in the pathogenesis of PD.

## 1. Introduction

Parkinson's disease (PD) is a progressive neurodegenerative disease that occurs in approximately 1% of the population over the age of 65 years. There are two types of PD, familial and sporadic forms of PD. Although familial PD cases account for 10% of total cases of PD, investigations of the functions of familial PD gene products have provided great insights into the molecular mechanisms of the onset of PD, and familial PD gene products are thought to also play roles in the pathogenesis of sporadic PD (see recent reviews [1, 2]).

The *DJ-1* gene has been identified by us as a novel oncogene that transforms mouse NIH3T3 cells in cooperation with activated *ras* in 1997 [3]. In 2003, Bonifati et al. found a large deletion and missense mutation in the *DJ-1* gene in Italian and Dutch PD patients, leading to identification of the *DJ-1* gene as a causative gene for familial PD *park7* with recessive inheritance [4]. Twenty-three pathogenic deletion and point mutations were found in patients with PD (see Parkinson's disease mutation database and references therein, http://www.molgen.ua.ac.be/PDmutDB/default.cfm?MT=0-&ML=0&Page=Home). Compared to *parkin* and *Pink1*, other causative genes of familial PD with recessive inheritance, the number of mutations in the DJ-1 gene is small; numbers of mutations of the three genes are the order of *parkin *> *Pink1 *>  *DJ-1*. This might be due to the position of DJ-1 during the course of onset of PD; DJ-1 may be placed upstream of *Pink1* and *parkin* [1, 2].

In this review, we describe functions of DJ-1 against oxidative stress and discuss how loss of function of DJ-1 affects the pathogenesis of PD.

## 2. Structure, Expression, and Function of DJ-1

DJ-1 is comprised of 189 amino acids with seven *β*-strands and nine *α*-helices in total and is present as a dimer [5–9]. Amino acid sequences of DJ-1 are conserved from prokaryotes to eukaryotes and they are now named DJ-1 superfamily [10]. DJ-1 is structurally most similar to the monomer subunit of protease I, the intracellular cysteine protease from *Pyrococcus horikoshii* [5, 11]. DJ-1, however, contains an additional *α*-helix at the C-terminal region, which blocks the putative catalytic site of DJ-1 [5–7].

DJ-1 is expressed in almost all cells and tissues, including the brain [3]. DJ-1 is expressed in both neurons and glia cells [4, 12, 13]. The expression level of DJ-1 is increased in cells, including reactive astrocytes, under an oxidative stress condition [14], and overexpression of DJ-1 is observed in reactive astrocytes in sporadic PD and other neurodegenerative diseases [12, 15–17]. Knockdown or knockout of DJ-1 expression in astrocytes impairs astrocyte-mediated neuroprotection against oxidative stress through deregulation of mitochondrial complex I and inflammatory responses [17–20]. 

**Figure 1:**
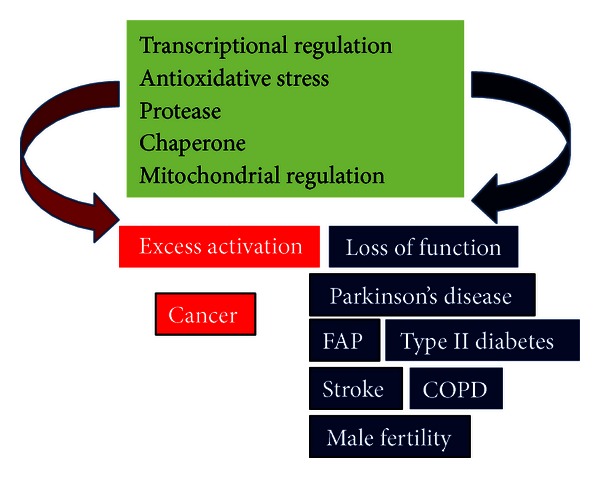
Functions of DJ-1 and its related diseases. DJ-1 is a multifunctional protein. It is thought that excess activation and loss of function of DJ-1 trigger the onset of various diseases, including cancer and Parkinson's disease.

**Figure 2:**
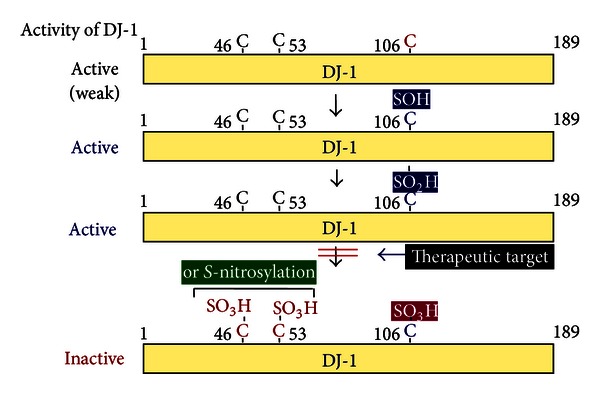
Cysteine oxidation and activation of DJ-1. DJ-1 contains three cysteine residues at amino acid numbers 46, 54, and 106 (C46, C54, and C106, resp.). C106 is sequentially oxidized with SOH, SO_2_H, and SO_3_H, and then C46 and C54 are oxidized or *S*-nitrosylated.

**Figure 3:**
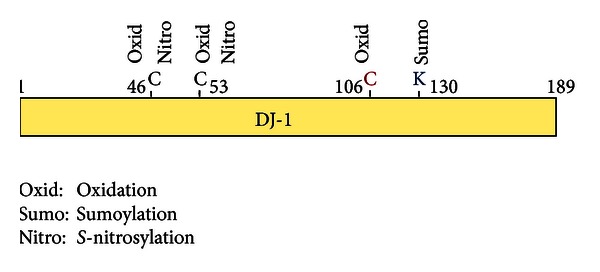
Posttranslational modifications on DJ-1. DJ-1 is oxidized at amino acid numbers 46, 54, and 106 (C46, C54, and C106, resp.), *S*-nitrosylated at C46 and C54 and sumoylated at K130.

DJ-1 is a multifunctional protein that participates in transcriptional regulation [21–29], antioxidative stress reaction [14, 30–36], and chaperone [37, 38], protease [39–41], and mitochondrial regulation [33, 42–52] (Figure 1). DJ-1 is located in the cytoplasm, nucleus, and mitochondria in cells, and secreted DJ-1 has been observed in various cultured cells and tissues, including cancer cells and tissues [14, 40, 53–60] and astrocytes [14]. DJ-1 is translocated from the cytoplasm to nucleus upon exposure to growth factors [3], and oxidation of C106 described later is necessary for nuclear translocation of DJ-1 [61]. DJ-1 contains three cysteine residues, C46, C56, and C106. Of the three cysteine residues, C106 is highly susceptible to oxidative stress and is oxidized as SOH, SO_2_H, and then SO_3_H [30, 33, 34], and mutation of C106 results in loss of all of DJ-1's functions [32, 33, 35] (Figure 2). DJ-1 at C106 with SO_3_H is thought to be an inactive form of DJ-1 [38], and excessive oxidized DJ-1 has been observed in brains of patients with PD and Alzheimer's disease [15, 62]. DJ-1 thus possesses quenching activity against reactive oxygen species (ROS) by self-oxidation of its cysteine residues [32, 63]. Phylogenetic analyses showed that, of the DJ-1 superfamily from prokaryotic and eukaryotic representatives, C106 is highly conserved and important for their functions, including enzymatic activities such as thiamin biosynthetic enzymes, protease and isocyanide hydratase, chaperone, and stress response ([10, 64], references therein). DJ-1 is also modified by sumoylation [65], *S*-nitrosylation [66], and phosphorylation [67] (Figure 3). Sumoylation of DJ-1 occurs under an oxidative stress condition in concomitant with acidic shift of DJ-1. Sumoylation of DJ-1 at lysine 130 is necessary for its activity, and excess sumoylation is observed in an L166P pathogenic mutant of DJ-1 [65]. *S*-nitrosylation is observed at cysteines 46 and 53 of DJ-1 under a nitrosative stress condition and affects dimerization of DJ-1, which is necessary for DJ-1 to exert its function [66]. DJ-1 is also phosphorylated in a p53-dependent manner, but phosphorylated amino acid(s) and the effect of phosphorylation on DJ-1 function are not known [67]. From these points, it is thought that DJ-1 also participates in the pathogenesis of sporadic PD as well as familial PD.

## 3. Transcriptional Regulation of DJ-1 in Response to Oxidative Stress and Dopamine Synthesis

Although DJ-1 does not directly bind to DJ-1 [68], it regulates the activity of DNA-binding transcription factors as a coactivator or corepressor through binding to DJ-1-binding transcription factors. Transcription factors whose activity is regulated by DJ-1 include the androgen receptor [21, 22, 27], polypyrimidine tract-binding protein-associated splicing factor (PSF) [25], p53 [23, 28, 69, 70], nuclear factor erythroid-2-related factor 2 (Nrf2) [26], and sterol regulatory element binding protein (SREBP) [68]. Considering oxidative stress response and dopamine synthesis, regulation of Nrf2, p53, and PSF by DJ-1 is important. Nrf2 is a master transcription factor for oxidative stress and detoxication responses. Without such stresses, Nrf2 is localized in the cytoplasm in a complex with Keap1, resulting in degradation by the ubiquitin-proteasome system. Upon oxidative stress, DJ-1 sequesters Keap1, leading to translocation of Nrf2 into the nucleus to activate various antioxidative stress genes, thereby decreasing the ROS level [26]. p53 is a tumor suppressor and plays roles in induction of senescence and apoptosis in cells and in regulation of mitochondrial homeostasis against oxidative stress. DJ-1 directly binds to p53 and regulates p53 activity in various ways; p53 is activated by Topors-mediated sumoylation and inactivated by DJ-1 through inhibition of Topors activity [23], and DJ-1 binds to the DNA-binding region of p53 to inhibit p53 transcriptional activity when affinity of p53 or its mutants to DNA is low, leading to cell cycle progression [70]. It has also been reported that DJ-1 inhibits the induction of apoptosis by p53-induced Bax expression [69]. DJ-1 stimulates the expression of superoxide dismutase (SOD 3) and glutathione ligase genes by an unknown mechanism to reduce ROS level [71, 72].

**Figure 4:**
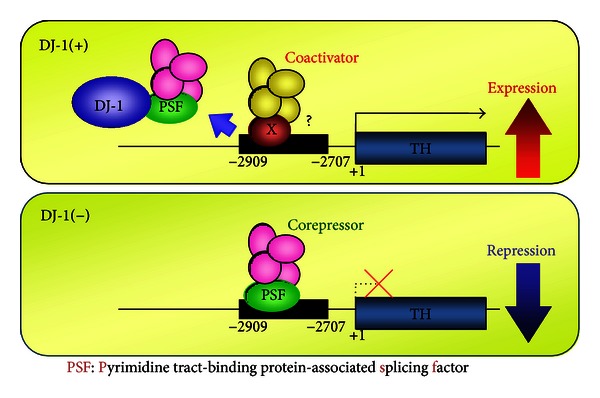
Schematic model of activation of the tyrosine hydroxylase gene by DJ-1. In the absence of DJ-1, PSF binds to the promoter region spanning −2909 to −2707 of the tyrosine hydroxylase (TH) gene to repress its transcription. In the presence of DJ-1, DJ-1 binds to PSF to sequester PSF from the TH gene, resulting in replacement of the corepressor complex with a coactivator complex, thereby activating TH gene transcription.

**Figure 5:**
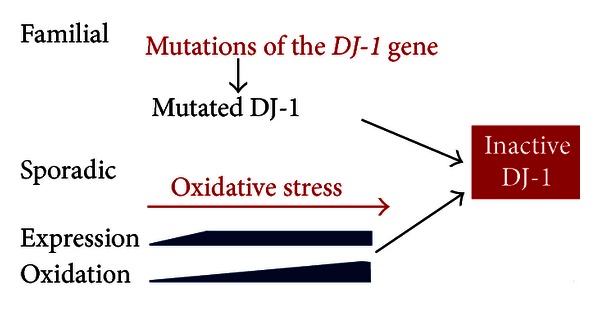
Proposed model of the role of DJ-1 in the onset of Parkinson's disease. In the case of familial Parkinson's disease (PD), the DJ-1 gene is heritably mutated, giving rise to inactive DJ-1 that causes PD. In the case of sporadic PD, DJ-1 expression is induced in cells upon oxidative stress to prevent cell death. During the course of continuous oxidative stress, DJ-1 is highly oxidized, giving rise to inactive DJ-1 that causes PD.

Dopamine is synthesized from tyrosine by two enzymes: tyrosine hydroxylase (TH) converts tyrosine to L-DOPA and L-DOPA carboxylase (DDC) converts L-DOPA to dopamine. Dopamine is then packed in synaptic vesicles by vesicular monoamine transporter 2 (VMAT2). Although TH level in PD patients is decreased, it is not changed in DJ-1-knockout mice [73–75]. DJ-1 positively regulates human TH gene expression by sequestering transcriptional repressor PSF from the human TH gene promoter [25] (Figure 4). This upregulation is only observed in the human TH gene due to lack of the PSF-recognition sequence in the mouse TH gene, and highly oxidized DJ-1 loses this activity [29]. This finding indicates one of the reasons for no change in TH level in DJ-1-knockout mice. DJ-1 also regulates enzymatic activities of TH and DDC [76]. When the sum of SH (reduced) and SOH forms of C106 is more than 50% of total forms of C106, DJ-1 upregulates TH and DDC activities, suggesting that the activity of DJ-1 toward TH and DDC is changed depending on the level of oxidative stress and that it is decreased with aging, which is one of the crucial factors for onset of PD (Figure 5). DJ-1 positively regulates expression of the VMAT2 gene and VMAT2 activity through transcriptional coactivator and protein-protein interaction, respectively [77]. Since VMAT2 re-uptakes excess dopamine into synaptic vesicles to prevent neurons from oxidized dopamine-induced damage, upregulation of VMAT activity by DJ-1 contributes to this reaction. Pathogenic mutations of DJ-1, including both homozygous and heterozygous mutations, have reduced stimulating activity against TH, DDC, and VMAT2 [29, 76, 77].

## 4. Chaperone and Protease Activity of DJ-1

Structures of DJ-1,* Escherichia coli* chaperone Hsp31 and an Archaea protease are conserved [7]. DJ-1 inhibits the aggregation of *α*-synuclein under an oxidative condition by its chaperone activity [37, 38]. As stated in Section 2, the structure of DJ-1 is similar to that of cysteine protease from *Pyrococcus horikoshii*, but C-terminal *α*-helix 9 blocks a catalytic domain of protease [5]. DJ-1 in dopaminergic cells undergoes C-terminal cleavage in response to mild oxidative stress, and a C-terminally cleaved form of DJ-1 with activated protease activity enhances cytoprotective action against oxidative stress-induced apoptosis [41]. Although protease activity is still in debate, low protease activity of DJ-1 has been reported [39–41]. Transthyretin, a causative protein in familial amyloidotic polyneuropathy (FAP), is degraded in cells transfected with full-sized DJ-1 and *in vitro *by recombinant DJ-1 lacking *α*-helix 9, and mutation of C106 in DJ-1 results in loss of its protease activity [40]. Localizations of DJ-1 and amyloid plaque of transthyretin in FAP patients are mirror images [40], and faint staining of DJ-1 is observed in the outer halo of Lewy bodies in PD patients [15]. These results suggest that *α*-helix 9 of DJ-1 is opened in cells under oxidative stress conditions in which oxidized protein(s) begins to aggregate and that DJ-1 degrades an aggregated protein(s) that causes neurodegenerative diseases. Identification of the recognition sequence of DJ-1 protease and of the protein(s) that opens *α*-helix 9 of DJ-1 in cells will lead to elucidation of the physiological role of DJ-1 protease.

## 5. DJ-1-Mediated Signaling Pathways against Oxidative Stress

There are several pathways against oxidative stress and these pathways prevent cell death, thereby leading to cell growth. The phosphoinositide 3-kinase (PI3 K)/Akt pathway is the major growth signaling pathway. When cells receive growth signals such as epidermal growth factor (EGF) stimulation, PI3 K triggers phosphorylation of Akt/protein kinase B (PKB), leading to activation of continuous phosphorylation cascades, resulting in stimulation of cell growth (see reviews [78, 79] and references therein). Phosphatase and Tensin homolog deleted from chromosome 10 (PTEN) is a lipid phosphatase that inhibits PI3 K and acts as a negative regulator of the PI3 K/Akt pathway. DJ-1 directly binds to PTEN to inhibit its enzymatic activity [80, 81]. After oxidative stress such as that caused by injection of a neurotoxin into mice or by addition of a neurotoxin to cultured cells, the Akt pathway is activated concomitantly with inactivation of PTEN in mouse brains and cultured cells, and the phosphorylation level of Akt is reduced in DJ-1-knockout mice, leading to neuronal cell death [80–82].

Apoptosis signal-regulating kinase 1 (ASK1) is mitogen-activated protein kinase-kinase-kinase 5 (MAP3 K5). It activates c-Jun N-terminal kinase (JNK) and p38 mitogen-activated protein kinases in response to various stresses such as oxidative stress, endoplasmic reticulum stress and calcium influx. TNF-*α*, LPS, and ischemia also trigger the generation of ROS, resulting in activation of ASK1 (see reviews [83, 84], references therein). ASK1 has been found to be involved in cancer [85, 86], diabetes [87, 88], and cardiovascular diseases [88–90] and neurodegenerative diseases [91–93]. These phenomena are similar to those observed in DJ-1-mediated diseases [3, 4, 40, 94–99]. The generation of ROS is also crucial for TNF-*α*-induced signaling pathway that leads to apoptosis, and treatment of cells with antioxidants such as N-acetyl-l-cysteine (NAC) inhibits apoptosis induction [83]. Daxx, a death domain-associated protein, associates with ASK1 in the cytoplasm to induce apoptosis after cells are treated with TNF-*α* [83, 84]. DJ-1 binds to both Daxx and ASK1 to sequester Daxx into the nucleus, preventing Daxx from association with ASK1, thereby inhibiting oxidative stress-induced apoptosis in H_2_O_2_-treated cultured cells and MPTP-administered-PD model mice [100, 101]. Pathogenic mutants of DJ-1 do not have this activity [102].

The ERK pathway is the main cell-progression pathway starting from Ras, followed by Raf, Mek, and ERK. DJ-1 protects against dopamine toxicity through the Erk kinase pathway in which DJ-1 and Erk are mutually activated upon administration of dopamine into mice or cultured cells [103]. It has been reported that an accelerated loss of substantia nigra cell bodies containing dopamine neurons was observed in aging mice lacking DJ-1 and the glial cell line-derived neurotrophic factor receptor Ret and that DJ-1 interacts with ERK signaling [104]. Furthermore, DJ-1 protects dopaminergic neurons against rotenone-induced apoptosis by enhancing ERK-dependent mitophagy [105]. Thus, DJ-1 prevents cells from oxidative stress-induced death by regulating various signaling pathways.

## 6. Role of DJ-1 in Mitochondrial Homeostasis

Mitochondrial dysfunction, including reduced mitochondrial complex I activity and mitochondrial membrane potential, is observed in PD patients [106–110] and in DJ-1-knockout mice and flies [47, 111]. Fragmented mitochondria are observed in DJ-1-knockout mice and cells [46, 48, 51]. Although a portion of DJ-1 is present in mitochondria under normal conditions [45, 112] and DJ-1 binds to subunits of mitochondrial complex I to regulate its activity [45], the translocation of DJ-1 into mitochondria is stimulated by oxidative stress, and oxidation of C106 with SO_2_H and N-terminal 12 amino acids is necessary for mitochondrial translocation of *DJ-1* [33, 113]. Pathogenic DJ-1 mutants such as L166P and M26I DJ-1 are localized in mitochondria as monomers [113]. DJ-1 ectopically targeted to mitochondria by the addition of an N-terminal mitochondrial targeting sequence has been shown to be more protective against oxidative stress-induced cell death [44]. Considering these findings, it is thought that localization of DJ-1 as a dimer in mitochondria is required for DJ-1 to play a role in antioxidative stress reaction and that DJ-1 localized in mitochondria as a monomer, such as M26I and L166P DJ-1, is, in contrast, harmful to cells. 

DJ-1 has no mitochondria-targeting sequence and binds to several chaperones, including Hsp70, CHIP, and mitochondrial Hsp70/mortalin/Grp75, suggesting that translocation of DJ-1 into mitochondria relies on or depends on other proteins, including mortalin [43]. Mortalin plays a central role in mitochondrial homeostasis through its capacity to direct the import of nuclear-encoded proteins carrying an internal mitochondrial targeting sequence into mitochondria, and mutations of the mortalin gene were found in patients with Parkinson's disease [114]. 

The role of DJ-1 in autophagy is still in debate, and almost all of the reports focused on mitochondria-specific autophagy, mitophagy. When mitochondrial membrane potential is decreased, DJ-1 is translocated into mitochondria to induce mitophagy, which is clearance of damaged mitochondria [48, 50, 52]. DJ-1 seems to act in parallel to the Pink1/Parkin-mediated mitophagy pathway [50]. Although mitochondrial functions of DJ-1 have been extensively studied, the precise mechanism of mitophagy induction by DJ-1 is still poorly understood.

## 7. Conclusion and Perspective

DJ-1 has multiple functions and plays a protective role against oxidative stress-induced cell death by using all of its functions. DJ-1 is also a stress sensor and its expression is increased upon various stresses, including oxidative stress. Loss of function and reduced function of DJ-1 trigger the onset of oxidative stress-related diseases, including Parkinson's disease [4, 94, 95], stroke [96, 97], familial amyloidotic polyneuropathy [40], chronic obstructive pulmonary disease (COPD) [98], and type II diabetes [99]. The oxidative status of C106 of DJ-1 determines all of the functions of DJ-1. Excess oxidation of C106 renders DJ-1 inactive, and highly oxidized DJ-1 has been observed in patients with Parkinson's disease and Alzheimer's disease. These results suggest that block of excessive oxidation of DJ-1 is a therapeutic target for the oxidative stress-related diseases stated earlier. Indeed, DJ-1-binding compounds that bind to the C106 region of DJ-1 showed neuroprotective activity against neurodegeneration in Parkinson's disease and stroke animal models through inhibition of excessive oxidation of C106 of DJ-1 [115–117]. 

## Abbreviations

PD: Parkinson's disease
PSF: Polypyrimidine tract-binding protein-associated splicing factor
ROS: Reactive oxygen species
Nrf2: Nuclear factor erythroid-2 related factor 2
TH: Tyrosine hydroxylase
DDC: L-DOPA carboxylase
VMAT2: Vesicular monoamine transporter 2
PTEN: Phosphatase and Tensin homolog deleted from chromosome 10
ASK1: Apoptosis signal-regulating kinase 1.
